# The Role of the Patients’ Companion in the Transitional Care from the Open Heart Surgery ICU to the Cardiac Surgery Ward: A Qualitative Content Analysis

**DOI:** 10.30476/ijcbnm.2021.87898.1475

**Published:** 2021-04

**Authors:** Sahar Khoshkesht, Shahrzad Ghiyasvandian, Maryam Esmaeili, Masoumeh Zakerimoghadam

**Affiliations:** 1 Department of Medical-Surgical Nursing, School of Nursing and Midwifery, Tehran University of Medical Sciences, Tehran, Iran; 2 Nursing and Midwifery Care Research Center, School of Nursing and Midwifery, Tehran University of Medical Sciences, Tehran, Iran

**Keywords:** Cardiac surgery, Family care giver, Intensive care unit, Transitional care

## Abstract

**Background:**

The patients’ companions can help improve transitional care as an important missing link, but their role is not clear. The aim of this study was to explore the role of the patients’ companion in the transitional care from the open heart surgery intensive care unit (OH-ICU) to the cardiac surgery ward.

**Methods:**

This was a qualitative descriptive study using conventional content analysis that was conducted from September 2019 to February 2020 in Tehran, Iran. Purposeful sampling method with maximum variation was performed among the patients eligible for transfer to the cardiac surgery ward, their companions, nurses, and physicians in charge of transferring from the OH-ICU to the ward. 27 in-depth and semi-structured interviews were conducted with 25 participants , and data were analyzed using the Granheim and Lundman method. The data were managed using the MAXQDA software (version 10.0).

**Results:**

Based on our analysis, the theme “Dual role of the patients’ companion” and its two categories, “Companion as a facilitator” and “Companion as an inhibitor”, were extracted. Emotional support, satisfaction of basic needs, care arm, alarm bell, and communication bridge were the sub-categories of the first category, and Interfering with care and creation of tension were those of the second category.

**Conclusion:**

We concluded that the patients’ companions can play an important role in transitional care, especially as emotional support and system assistants due to the structure of the health care system and Iranian cultural context . Therefore, it is suggested that the patients’ companion should be considered as a member of the transition team and accompany them in this process by informing and supporting them.

## INTRODUCTION

Coronary artery disease is the most common heart disorder and cause of death. ^1^ Coronary artery bypass grafting (CABG) is a life-saving treatment in these patients. ^2^ Following this surgery, patients need monitoring and are usually hospitalized in the open heart surgery intensive care unit (OH-ICU) for 48-72 hour. Transferring a patient from the OH-ICU to the general ward is done so that the patient care can be continued and is a risky and challenging process. ^3 , 4^ Recently, the use of Liason nurse services for safe and effective transfer is expanding, but there is no liaison nurse service in medical centers in Iran. ^5^ Therefore, the patient’s companions as a permanent member in the transfer process can play an important role in the transition. ^6^ However, they are not involved in planning transitional care programs and there is not much information about the way they affect the transfer process, their needs and wants, and their level of involvement. ^7^ The nurses as fasilitator of communication should perceive the companion’s roles and provide them with services in the transitional care team. ^8^

The patients’ family caregivers have become silent care coordinators. Besides, they provide direct nursing and supportive care, facilitate communication, identify warning signs, identify the patients’ strengths and weaknesses, identify their needs and preferences, are the patient’s advocates, and help the patient make decisions. Family caregivers play an important role in supporting the patient during the transition and contribute to the continuity of care. ^9^ The patients’ companions, who are usually family members of the patients, play an important role in caring for their loved ones during the transfer. ^10^ A qualitative study that examined the experiences and roles of caregivers in facilitating the transfer of patients from the orthopedic ward to the rehabilitation ^7^ and another study conducted in Brazil, stressed the importance of the quality of communication between the patient and the family and care providers to the continuity of care during the transition. ^6^

According to the complexity of patient care during the transition from OH-ICU to the general ward, the patient’s companion as an important missing link can help to improve the transitional care. Some studies have examined the experience of patients and their families during the transfer ^11 , 12^ and many efforts have been made to integrate the role of the patient’s companion as a treatment partner in the delivery of transitional care. ^13 , 14^ Also, in Iran, some studies have examined the experiences of the families and needs of patients hospitalized in ICU. ^15 , 16^ However, due to the impossibility of presenting the companions in the ICU or the general ward in Iran, cultural differences and attitudes, the role of the patient’s companions, and their level of involvement, especially in the transitional care, is not clear and none of these studies has addressed such perceptions. Therefore, the aim of this study was to explore the role of the patients’ companion in the transitional care from the OH-ICU to the cardiac surgery ward.

## METHODS

This is a qualitative descriptive study using a content analysis method that was conducted from September 2019 to February 2020 in Tehran, Iran. The method of content analysis which is used to provide an objective, systematic, reproducible description of the manifest content of the message and identify the concepts and categories of the latent message was appropriate to the nature of this research. ^17^

The study participants were selected using purposeful sampling method with maximum variation (age, sex, level of education, social, economic, cultural status) from Imam Khomeini hospital affiliated to Tehran University of Medical Sciences. The following inclusion criteria were considered to choose the sample of this study. These criteria for patients were volunteer patients under CABG eligible for transfer according to the physicians’ order, with full consciousness and without a history of advanced chronic disease, emergency, or unpredictable conditions. These criteria for the medical team were volunteer nurses and physician in charge of the patient transfer and admission in both OH-ICU and cardiac surgery ward with at least 6 months of work and patient transfer experience. We also interviewed the persons who accompanied the patients in the transfer process as their companion. The only exclusion criterion was the lack of willingness to continue the study. Since the number of participants in the qualitative study was not predictable, sampling continued until data saturation. Based on these criteria, a total of 25 participants (11 patients, 5 companions, 4 nurses from ICU and 4 nurses from cardiac surgery ward, and 1 physician) were enrolled in the study. 

The data were collected through 27 in-depth and semi-structured interviews by S.Kh and M.Z as researchers. All interviews were recorded and lasted from 15 minute s to 1 hour. Because of instability and probability of forgetfulness in ICU patients due to medications effects and to ensure that the participants could recall their experiences, all interviews were conducted after stability in the first week after transfer on the patient’s bedside in a quiet place in cardiac surgery ward. It should be noted that interviews with two patients were repeated in different situations (before transition in the ICU and after transition in cardiac surgery ward) to ensure all aspects of transfer were covered. The interview was based on guiding questions and was completed using probing questions such as “What do you mean”, “Can you explain more”. The main questions when interviewing physicians and nurses were: “How do you see the role of the patient’s companion in the transition process of the patient to the general ward?” Another question was: “Based on your experience, how can the companion participate in the transitional care program?”, and “What barriers do you see in involving the companions in the transitional care?” In an interview with the patients and companions, the first question was: ”Please, describe your experience when transferring a patient from OH-ICU to general ward”; the other question was: “What was your position in the transitional care of the patient to the general ward? “ It should be noted that a companion means any member of the family, partner, or friend with whom the patient feels comfortable and has accompanied him/her in the process of hospitalization until transfer to the general ward and discharge from the hospital. Data analysis was performed based on Granheim and Lundman’s method (2004). ^18^ First, to receive a general understanding of the content, we transcribed the recorded interviews and read them several times. Then, they were considered as a unit of analysis. In the next stage, the words, sentences, or paragraphs that contained aspects related to each other were considered as meaning unit. Then, the meaning units were condensed. The process whereby the condensed text is abstracted on a higher logical level has been called abstraction that include the creations of codes, categories, and themes. In the next stage, the concepts hidden in the meaning units were extracted and labeled as code. Then, all the codes were compared, and the conceptually similar codes were categorized. After reviewing and gaining consensus by the research team, they generated clear definitions and names for the theme. ^18^ It should be noted that software MAXQDA (version 10.0) was used to manage and organize the data.

The following measures were taken to ensure the rigor and trustworthiness based on the proposed criteria of Goba and Lincoln. ^19^ Sampling was done with maximum variety. The continuous presence of the researcher in the field and communication with the participants, long-term engagement, immersion in the data , and member check were considered to increase the credibility. To approve confirmability, all authors checked all the codes and themes. To get dependability, we kept the same questions for data collection. We also tried to explain all the processes of research precisely and attended audit trials to gain transferability. It should be noted that we followed the consolidated criteria for reporting qualitative studies (COREQ). ^20^

This study was approved by the Joint institutional research ethics Committee of School of Nursing and Midwifery &amp; Rehabilitation, Tehran University of Medical Sciences (IR.TUMS.FNM.REC.1398.020). Written and orally informed consent was taken from all the participants. The subjects were informed about the aims of the study. The researcher assured them that anonymity would be guaranteed, attendance was voluntary and they could withdraw from the study whenever they wished, and attending this study would not interfere with their treatment process. Also, written permission was taken to record the interviews. 

## RESULTS

In this study, 25 participants consisting of patients (in transition, the first, second, third, and fourth day after transfer and the last day of hospitalization), companions (relatives), nurses (with 1 to 28 years experience), and physician (surgeon and transfer manager) were interviewed. The other characteristics of the participants are summarized in Table 1. 

**Table 1:** Demographic characteristics of the participants (N=25)

Participant number		Age (years)	Gender	Educational level	Occupation
1	Patient	30	Female	Elementary	Housewives
2		74	Female	Elementary	Housewives
3		60	Female	Elementary	Housewives
4		75	Female	Elementary	Housewives
5		35	Male	Bachelor	Employees
6		53	Male	Elementary	Farmer
7		51	Male	Doctor	Physician
8		63	Female	Elementary	Housewives
9		50	Male	Bachelor	Employees
10		61	Female	Elementary	Housewives
11		63	Male	Diploma	Retired
12	Companion	49	Female	Elementary	Housewives
13		50	Male	Bachelor	Employees
14		33	Female	Bachelor	Housewives
15		30	Female	Diploma	Housewives
16		35	Male	Bachelor	Employees
17	Medical team	34	Female	Bachelor	ICU nurse
18		44	Male	Bachelor	ICU nurse
19		45	Male	Bachelor	ICU nurse
20		60	Female	Bachelor	ICU and general nurse
21		42	Female	Bachelor	ICU nurse
22		26	Female	Bachelor	General nurse
23		47	Female	Bachelor	General nurse
24		24	Female	Bachelor	General nurse
25		53	Male	Assistant Professor	Cardiac surgeon

Dual role of the patient’s companion appeared as a theme, including companion as a facilitator and companion as an inhibitor as categories with 7 sub-categories (Table 2). 

**Table 2:** Sub-categories, categories, and theme of the study

Sub-categories	Categories	Theme
Emotional support	Companion as a facilitator	Dual role of the patients’ companions
Satisfaction of basic needs		
Care arm		
Alarm bell		
Communication bridge		
Interfering with care	Companion as an inhibitor	
Creation of tension		

### Dual Role of the Patients’ Companion

The dual role of the patients’ companion in the transitional care that emerged in this study means that while they can be helpful, they can also interfere with the treatment and care. In the Iranian health system, the patient’s companions have always been used as system assistants. Although this accompaniment is not accepted by the staff due to overcrowding, increased risk of infection, stress , and interference in care, the benefits of their presence are used where necessary. According to the results of this study, the patients needed the presence of the family in the transfer process. However, most nurses acknowledged that while the companion could be helpful, it was just as much an inhibitor. 

For example, a nurse participant said, *“I think the patient’s companions’ role is 50-50, 50% can be very helpful and 50% can be ineffective”*. (P18) While another nurse said. *“I feel you should not follow what is said in the books. In our cultural context, I did not see a good result from participation of the companions in transitional care; they do not know anything at all”*. (P 19)

Therefore, it seems that although nurses have accepted the presence of the family for cultural reasons, they have not understood the role of the family or their influence as a member of the transfer team and in the transitional care. Therefore, the role of the patient’s companion was classified into facilitators and inhibitors. 

### 1- Companion as a Facilitator

Companion as a facilitator means the patient can play an intermediary role in the transitional care (before transfer in the ICU, during transfer, and after transfer in the general ward) to help continuity of care, which will be described below.

### 1-a. Emotional Support

The emotional support role of companions was emphasized by the patient and the family, as well as the medical team. Hospitalization, surgery, ICU stays and transition to the general ward are the stressful facts that can cause anxiety and emotional tensions. Therefore, one of the most important needs of these patients is emotional support. Receiving support and peace from a person who is familiar and empathetic and has emotional desires will be helpful.

A patient participant believed that *“Companions can satisfy emotional needs. Sometimes when I hold the hand of my child, energy comes and circulates in my body. It is not a professional issue it’s an emotional one.”* (P 7)

A physician also said *“Presence of companions in the ward is very helpful psychologically because the soul, mind, and body are connected”*. (P 25)

The patient’s companion with emotional support can also be effective in improving his treatment process, act as a catalyst for recovery, and facilitate the healing process. A nurse participant believed: *“After the operation, the family plays a key and main role in the recovery of the patients”*. (P 8)

### 1-b. Satisfaction of Basic Needs 

In cardiac surgery ward, due to shortage of staff, it is not always possible to use the crew to help meet the patient’s basic needs. Therefore, the patient’s companion is helpful in meeting the needs of the patient. Some nurses believed that the companion was only helpful in meeting the patient’s primary needs. However, addressing this need from the patients’ point of view had another aspect. Some patients need to have companions due to embarrassment, lack of awareness of their rights, or fear of abuse. Some others believed that helping patients to meet basic needs was not part of the nurses’ job duties. Of course, the shame caused by Iranian and Islamic culture was highlighted. A patient participant believed that *“For example, if you want to go to the bathroom, he (companion) helps. The staff also helps, but you can’t face it,”* (P 8), and another patient said, *“Suppose I want something. I am ashamed of it. I can’t say. There is some staff who may say it’s not our duty; they grumble”*. (P 7)

### 1-c. Care Arm

Companion, as a care arm, was another role in this study. Some companions in our study were involved in providing direct care to their family members (e.g. management of cough, dyspnea, nutrition, exercise, medication). Also, as mentioned earlier, nurses use the companions’ assistance even during transfer and care in the cardiac surgery ward. In fact, in addition to helping to provide the patient with care, they function insteadof the nurse. 

A nurse who worked in the cardiac surgery ward said: *“if companions are alert and cooperate with us, I tell them to be careful that the patient coughs well, use a spirometer or raise his/her legs”*. (P 21) and another nurse who worked in the ICU believed that *“if the patients are not stable, we will make sure that their companions be present. Then, we leave the patient to them”*. (P 20) 

### 1-d. Alarm Bell

A companion, as an alarm bell, had another role. Companions played this role, due to the close relationship and the patient’s feeling of comfort in talking about the symptoms and problems with them. From the point of view of the medical staff, the effective role of the companions in recognizing the warning signs and unstable conditions of the patient, which may be hidden from the eyes of the nurses due to the impossibility of direct supervision and work pressure, was appreciated. However, natural symptoms may be identified as an alarm sign by the companion; conversely, they may not consider the warning signs to be important, which is related to the companion’s ignorance.

A nurse participant believed that *“If something happens to a patient, they can report it quickly. At the same time, the nurse cannot be at the bedside of 18 patients”* (P 23); one of the physicians said: *“I say that the role of the patient’s companion is very helpful in the ward, which means that if the patient’s condition does not go as well as expected towards a good recovery, the companion can inform the nurse. The nurse should also listen to them”*. (P 25)

One patient said, *“One of the patients had fallen out of bed. His companion quickly picked him up and informed the nurse”*. (P 2)

### 1-e. Communication Bridge 

Based on the results, the companions can be helpful in removing linguistic and cultural barriers, supporting the patient by facilitating the patient communication with the transitional team, and transmitting education to the patients. However, it seems that due to the non-demanding spirit that exists among the patients and companions in Iran, this relationship is one-way, so that only physicians and nurses referred directly to this issue. *“There are people who came from other cities. It is difficult to communicate with them. The family can inform us about the morals, characteristics, and habits of patients to us.”*.He also found the presence of the companion fruitful in convincing the patient, especially during the transfer. *“Presence of companions during the transfer can greatly reduce their anxiety. It means that if you justify the companions, they can easily convince the patient ”*. (P 19)

Sometimes, ICU nurses use companions for communication reasons. Some situations may include pediatric patients, persons who have language barriers, or those with mental illnesses who only communicate with certain people.

*“Most of our patients are old or do not speak Persian. We have difficulty speaking. Even if the patient is intubated and does not understand our language, we are calling their companion to cooperate whit us”*. (P 21)

Similarly, one of the companions said: *“For example, my patient is old and has come from a village. If she has a need, she should say to me; she can’t communicate with the nurses”*. (P 14)

### 2- Companion as an Inhibitor 

Companion as an inhibitor is when the patient interferes with the transitional care (before transfer in the ICU, during transfer, and after transfer in the public ward) and can disrupt or delay the care, as described below.

### 2-a. Interfering with Care 

 According to physicians and nurses, lack of knowledge, low level of education, and cultural differences are the reasons for interfering with care.

A nurse participant emphasized that *“I think it depends on their (companions) level of literacy. We have companions who are very old or illiterate. They think if the patient comes here, he just has to sleep. They may not let the patients walk or bath”*. (P 22)

Another nuse patritipant pointed out the role of culture besides the lack of awareness and said, *“Lack of awareness causes us a lot of trouble. There should be an interaction. If we are in the same direction, we will certainly progress, but it depends on the cultural level”*. (P 20)

### 2-b. Creation of Tension

Another inhibitor role of companions, especially in the ICU, was the creation of tension. The reason for this should be sought in the cultural context of Iran and the attitude of nurses about the companions. The tension between the nurse and companion can be created because of the lack of knowledge of companions and irresponsibility of nurses to answer their questions. 

A nurse who worked in the ICU believed that *“A 24-hour presence in the ICU may have many benefits, but I do not like it. It may cause tension between the nurse and the companion.”* (P 18) Another nurse said: *“I feel that the presence of the companions has not a good result due to our cultural conditions because they exaggerate. If the patient sighs, they also do so”*. (P 19). One the other hand, one of the companions belived that, *“In my opinion, if each patient has his/her companions, it will be crowded and causes tension. The hospital discipline will be disrupted”*. (P 16)

Most of the nurses also saw it as a reason to build resistance to the acceptance of attending companions in the ward by the medical staff. 

## DISCUSSION

In this study, the role of emotional support in the whole transfer process (in the ICU, during the transfer, and in the general ward) was expressed by all participants. A study used acronyms DECAF (D - Direct care provision; E - Emotional support; C - Care coordination; A - Advocacy; F - Financial) to describe the role of companions in the patient transfer process, which is largely in line with our study. ^14^ For example, emotional support was also observed in our study. It seems that by providing the opportunity for appointment through arranging meeting behind the ICU door, usingvideo monitors, communicating with the family by phone or video call can assist the emotional support of companions, decrease the patient’s anxiety, and improve recovery. Also, the void of the family can be partially compensated in the ICU with further support by nurses. In general wards, this can be followed by facilitating the possibility of engaging the companions or allocating time to follow their patients’ condition. During the transfer, the presence of a companion should be mandatory. The attendance of a companion reduces anxiety and increases the patient’s acceptance of the new conditions. In fact, it is possible to use the emotional supportive role of companions to reduce the patient’s anxiety and, consequently, reduce his/her physical problems and complications.

Participation in meeting the patients’ basic needs was another companion’s role along with considering the Iranian and Islamic culture. In addition, some companions in our study were involved in providing direct care to their family members (e.g. management of cough, dyspnea, nutrition, exercise, medication). This role was classified as the care arm. Although other studies have indicated the active role of companions in performing care, ^13 , 14^ adequate training was not performed by health care professionals. Consideration should be given to 2 questions. Whether the companion is sufficient to perform these tasks, and whether the caring role is normally performed by the nurse can be avoided under the pretext of lack of manpower and time or work pressure. It is recommended to define the boundaries of the tasks, train the companions, and ensure the relative competence of the companion in performing non-specialized tasks, and then to trust them and get their help. 

In the present study, the alarm bell was considered as the role of companions. The companions are in the first line to recognize the care problems. ^21^ In the previously mentioned study, although this role was not directly addressed, the guidance of the health system by the companions was emphasized. ^13^ In thatstudy, the transfer from the hospital to the rehabilitation center was considered, but in our study, more emphasis was placed on the patient’s warning signs in the hospital environment and in the absence of a nurse. However, whether the companions are aware of the alarm signs and express their request in a reasonable and appropriate manner should be considered. Therefore, it seems that training the companions regarding warning signs is suggested to be used as a safety valve in the care of the patient, but this presence should not affect the main responsibility of the nurse. The companion, as a bridge of communication, was another role in this study. This can be examined from two dimensions. One of them is cultural and linguistic barriers when the companions help the care providers to deliver care, and the other one is a point that can be inferred according to the Iranian culture and the non-demanding patients. Here, the companion role in conveying the patient’s needs and preferences is highlighted. In some articles, this role is manifested as the patient’s advocacy. ^13 , 14^ We did not specifically consider this role because it seems that in the cultural context of Iran, companions are also cautious in transmitting the patient’s preferences. In one critical review study, the role of the advocate and care coordinator was mentioned. ^13^ In the another study, the emphasis was on establishing a proper relationship with the companion for the continuation of care. ^6^ It seems that by establishing proper communication, we should also strengthen this bridge of communication, and convert it to a two-way free flow of communication. The inhibitor role of the companions was generally noted by nurses. These obstacles were described as interfering with care and creating tension. Despite the general positive attitude of Swedish nurses about the importance of families in nursing care, there was evidence of a negative attitude of newly graduated RNs ( registered nurses). ^22^ A study conducted on the nurses’ attitudes towards the importance of families in nursing care showed that they considered the caregivers as stress and a problem, and such factors as lack of time and the patient’s priority over the family caused them not to accept the companions’ presence. The results of the above study indicated that the cause of inconsistency in the companion’s role was lack of precise definition of the roles, an overlap of tasks, and unrealistic expectations. They concluded that the health professionals’ approach tocompanions was so contradictory and ambiguous. They considered them both a problem and a solution. ^22^ The present study included a small sample of companions and patients, which could limit the possibility of transferability. Nevertheless, the sample size was acceptable for achieving saturation. Also, this study was conducted on a single site that can be another limitation. On the contrary, to the best of our knowledge, this is the first time that a study explores the patients’ companion roles in all phases of ICU transition (before, during, and after) and includes the perspective of all persons involved in the transitional care. This is one of the major strengths of this study. Also, our data are in depth, rich, and well described.

## CONCLUSION

The findings of the present study highlighted the need to raise awareness of the transitional care team regarding the necessity of having communication with companions and benefits from their role to facilitate the transitional care. Our findings can be used to design a new transitional care program by involving the patients’ companions. Therefore, it is suggested that further studies should be carried out to evaluate the effect of involvement of the family in transitional care and assess their possible outcomes. 
